# Inflammation and Hypertension: Are There Regional Differences?

**DOI:** 10.1155/2013/492094

**Published:** 2013-03-21

**Authors:** Patricio López-Jaramillo, Carlos Velandia-Carrillo, Julie Álvarez-Camacho, Daniel Dylan Cohen, Tatiana Sánchez-Solano, Gabriela Castillo-López

**Affiliations:** ^1^Fundación Oftalmológica de Santander-Clínica Carlos Ardila Lulle (FOSCAL), Floridablanca, Santander, Colombia; ^2^Instituto de Investigación, Facultad de Medecina, Universidad de Santander (UDES), Bucaramanga, Santander, Colombia; ^3^Fundación Oftalmológica de Santander-Clínica Carlos Ardila Lulle (FOSCAL), Calle 155 A No. 23-09, El Bosque, Floridablanca, Santander, Colombia; ^4^Facultad de Medecina, Pontificia Universidad Católica del Ecuador (PUCE), Av. 12 de Octubre 1076 y Roca, 17 01 21 84 Quito, Ecuador

## Abstract

Hypertension is a chronic disease with global prevalence and incidence rapidly increasing in low and medium income countries. The surveillance of cardiovascular risk factors, such as hypertension, is a global health priority in order to estimate the burden and trends, to appropriately direct resources, and to measure the effect of interventions. We propose here that the adoption of Western lifestyles in low and middle incomes countries has dramatically increased the prevalence of abdominal obesity, which is the main source of proinflammatory cytokines, and that the vascular systemic inflammation produced by adipose tissue contributes to the development of hypertension. The concentration of proinflammatory cytokines is higher in the Latin American population than that reported in developed countries, suggesting a higher susceptibility to develop systemic low-degree inflammation at a given level of abdominal obesity. These particularities are important to be considered when planning resources for health care programs. Moreover, studying these singularities may provide a better understanding of the causes of the burden of cardiovascular risk factors and the remarkable variability in the prevalence of these medical conditions within and between countries.

## 1. Introduction

Hypertension is a chronic disease with global prevalence and rapidly increasing incidence in low and medium income countries, particularly in urban areas [1]. Indeed, while the prevalence of hypertension is decreasing in high income countries, it appears to be rising at alarming rates in low and middle income countries [2] where approximately 639 million people are living with hypertension. Moreover, in these countries a substantial proportion of hypertension is poorly controlled due to limited access to health services [1–7]. Clearly, the surveillance of cardiovascular disease risk factors such as hypertension is a global health priority, to estimate the burden and trends, to appropriately direct resources, and to measure the effect of interventions [2].

## 2. The Role of Hypertension in Cardiovascular Diseases

Considering that the main causes of mortality due to cardiovascular diseases (CVDs) include complications associated with hypertension, the lower awareness of this disease in low income countries is of great concern. Two recent large epidemiological studies that included low to middle income countries in Latin America, Asia, and Africa were conducted to determine the risk factors associated with a first myocardial infarction (the INTERHEART study [8]) and with a first stroke (the INTERSTROKE study [9]). These studies determined both global and region-specific population-attributable risk (PAR) for each major risk factor. Hypertension was identified as one of the primary risk factors for both outcomes, but it was also observed that particularly for stroke, the PAR associated with hypertension was substantially higher in low and middle income than in high income countries (Table 1).

It has been demonstrated in various animal models and from data in human twin and family studies that blood pressure is regulated by different genes [10, 11]. Nonetheless, many environmental risk factors are also associated with the development of hypertension. Factors associated with industrialization and urbanization such as obesity, high dietary salt intake, excessive alcohol consumption, social stress, and the ageing of the population are recognized as important contributory factors to the increases in blood pressure [5]. In low to middle income countries changes such as the increased access to westernized diets and the discontinuation of traditional dietary habits may have facilitated the expression of these pathologies and underlie the dramatic increases in the prevalence of hypertension observed in recent years [5].

## 3. The Role of Inflammation in Hypertension

Some years ago we demonstrated [12] that ultrasensitive C reactive protein (uCRP), a marker of low grade inflammation, was increased in individuals with hypertension. Based on these findings we hypothesized that low-degree inflammation could be an independent risk factor for essential hypertension [12], a proposal that has recently been reviewed and supported [13, 14]. 

**Figure 1:**
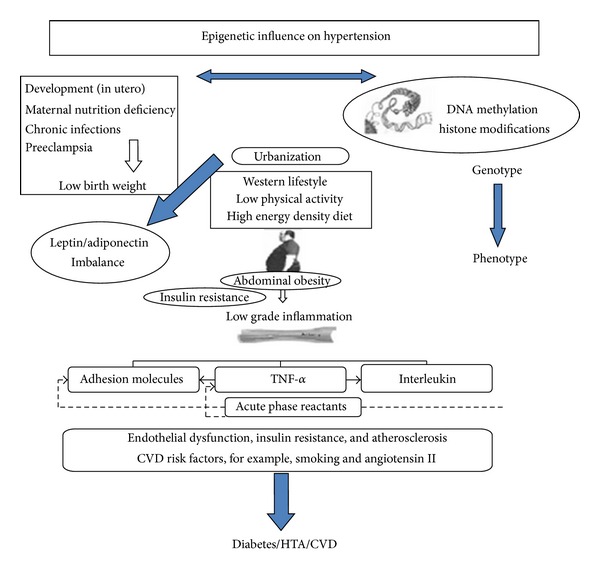
Early programming inducing stressors lead to alterations in gene expression (such as methylation of DNA or modification of histones) on phenotype producing persistent influences on metabolism. The conflict between the earlier programming and the later presence of abdominal obesity may have produced a higher sensitivity of this population to develop a state of low degree inflammation, insulin resistance and, consequently, an epidemic of hypertension, diabetes, and CVD.

As is shown in Figure 1, the process of urbanization and the adoption of Western lifestyles in low and middle income countries may play a role in the rise of abdominal obesity (AO) that has been described in these countries. Visceral fat is a relevant source of proinflammatory cytokines [15–17] which are significantly elevated in the serum of obese subjects [18]. It has been proposed that the vascular systemic inflammation produced by adipose tissue contributes to the development of hypertension, since inflammation produces endothelial dysfunction [19]. C-reactive protein (CRP), produced by the liver in response to the stimulus of tumor necrosis factor-alpha (TNF-*α*) and interleukin-6 (IL-6), is increased in subjects with multiple acute coronary events and is a strong independent predictor of new acute coronary events [20–22]. Moreover, we have demonstrated that in Andean women, CRP is an independent risk factor for pregnancy-induced hypertension [23, 24] and that in this population the concentration of CRP is increased in dyslipidemic subjects with MS [25, 26] as well as in overweight children [27].

Despite the differences in quantification methods and some disputed results, it appears that in general higher levels of proinflammatory cytokines are reported in non-Caucasians compared to Caucasians within the USA and UK (Table 2). These data support the proposal that there are ethnic differences in inflammatory markers that may also contribute to the variations in disease prevalence reported.

Recently, we conducted a study in schoolchildren in Bucaramanga, Colombia. The findings demonstrated a positive correlation between BMI, systolic blood pressure, and CRP [27]. These findings suggest that the correlation between CRP and hypertension previously reported in Colombian adults [12, 25] is also present among children and reinforces the idea that there is a link between adiposity (particularly visceral adiposity), increased BP, and increased plasma concentrations of inflammatory markers such as CRP. Several studies have reported ethnic differences in CRP and other proinflammatory cytokines concentrations in schoolchildren of developed and undeveloped countries (Table 3). Cook and colleagues [28] measured CRP concentrations in a representative sample of the population of England and Wales that included 699 children aged 10 to 11 years. That study showed that serum concentrations of CRP correlated positively with BMI, heart rate, systolic blood pressure, fibrinogen, and high-density lipoproteins, but not with other lipid fractions. Interestingly, it was found that the small number of children of South Asian origin had CRP levels 2.04 times higher than those from age, sex, and BMI-matched children. Ford [29] analyzed the results of the National Health and Nutrition Examination Survey of the United States (NHANES, 1999-2000), which included 2486 boys and girls aged between 3 and 17 years. BMI was the best predictor of CRP plasma concentration but the study also found ethnicity to be a determinant in boys aged 8 to 17 years and girls aged 8 to 11 years. Specifically, there was a higher CRP concentration in Mexican-American children in comparison with Caucasian-American children. In Colombia, we also found a significant positive correlation between BMI and CRP, and in accordance with higher levels of CRP in boys and girls from the upper tertile of BMI [27]. It is important to note that the CRP concentrations of the second tertile of BMI in Colombian children were as high as those reported in overweight and obese Caucasian-American and European children in a similar age range [29, 30].

On the basis of data from our study in children and previous studies in our adult population [12, 25, 26], it is interesting to propose that populations of low and middle income countries are predisposed to produce an inflammatory response at lower body fat levels than Caucasian populations. We suggest that the above is a consequence of a shorter time of exposure to the new lifestyles associated with modernization in these populations. We also propose that less exposure time leads to a delay in the adaptation process which results in a greater risk of low grade inflammation and insulin resistance at lower levels of abdominal obesity. Currently, poor hygiene (lack of potable water, defective waste, and sewage removal), high intestinal parasitism, infections, and tropical diseases are still present in Latin America. Superimposed on this proinflammatory background is the recent imposition of lifestyle habits that include diets high in saturated fats and refined flours, more smoking, increased physical inactivity, and abdominal obesity, all also associated with low grade inflammation [31, 32].

## 4. Why Are the Populations of Low and Middle Income Countries More Prone to Develop Low Grade Inflammation?

We suggest that this is a result of shorter exposure times among populations of developing countries to the new lifestyles associated with modernization. The shorter the exposure time, the less adapted the population is and the greater the risk of low-degree inflammation and insulin resistance at lower levels of abdominal obesity. Although the relative contribution of genetic, epigenetic, and environmental factors is not known, it is well documented that the Hispanic population in the USA and the South Asian population in the UK are at greater risk of low-grade chronic inflammation, DM2, and cardiovascular mortality than the Caucasian populations in those countries [33]. We have speculated that for minority populations who have recently migrated to high income countries from developing countries, the substantially shorter time of exposure to Western lifestyles is itself a risk factor for low grade inflammation. Data from the Pima Indians both exemplifies the influence of exposure to the Western lifestyle and demonstrates the greater susceptibility to these outcomes within indigenous populations with more recent exposure to it. The prevalence of both obesity and diabetes in the US Pima is substantially higher than in both their genetically similar counterparts living in Mexico and Caucasian Americans. In addition to genetic and environmental factors, intrauterine conditions and “epigenetic” influences are also thought to contribute to the elevated risk of obesity in the US Pima [34]. Nonetheless, further research is needed to specifically evaluate our hypothesis that the *length* of exposure to the “obesogenic” Western lifestyle modifies the association between obesity and inflammation.

## 5. The Role of Environment and Epigenetics in Hypertension

Therefore, the dramatic increase in incidence of hypertension in low and middle income countries may be associated with rapidly changing environmental conditions interacting with ethnic characteristics [14, 35]. Genetic predisposition associated with particular ethnic groups and lifestyle factors may also interact with in utero and early life conditions with respect to disease incidence. The *Developmental Origins of Disease* hypothesis emphasizes that there are critical periods in early life during which body structure and physiologic function are programmed for life. More recently, these effects of environment have been conceived in terms of epigenetics [35].

Epigenetics refers to functional alterations in gene expression or phenotype that do not change the underlying DNA sequence. These alterations induced by environmental conditions and mediated by modifications such as methylation of DNA or modification of histones can be transmitted to daughter cells thereby producing not only persistent, but also intergenerational influences on metabolism [36].

The mechanisms that control epigenetic processes are not completely understood, but it is clear that heritable DNA variation might alter the sensitivity to certain environmental triggers or change the nature of the epigenetic responses to a given exposure. In the Latin American context, the question is do regional and ethnic variations in epigenetic processes or simply differences in the environmental conditions explain the increased prevalence of hypertension?

Despite the increased prevalence of childhood and adult obesity in Latin America [32], maternal and childhood undernutrition remains a substantial public health problem within the region [32, 37]. While in children cardiovascular risk factors are strongly associated with BMI, somewhat paradoxically, a high prevalence of arterial hypertension is reported in stunted children and adolescents and adults within Latin America [38–41]. One study in Brazil [38] that investigated blood pressure in a random sample of adolescents who lived in slums and were exposed to nutritional stunting (10–16 years old, *n* = 56) showed that 51% had increased blood pressure and were at risk for hypertension. The prevalence of diastolic hypertension was 21% (95% CI = 10%–32%). The prevalence of cases with a systolic or diastolic arterial pressure above the 90th percentile was 51% (95% CI = 37%–65%). Another study conducted in the northeast of Brazil [39] included 416 adult slum residents and found hypertension in 28.5% of the population (women = 38.5%; men = 18.4%). They also observed that the height was associated with blood pressure, and in obese women lower height was associated with increased risk of hypertension (OR 1.98 95% CI 1.2–2.9). Another recent survey [40] investigated the association between height and health outcomes in mothers and offspring and found that short maternal height was independently associated with obesity, abdominal obesity, and increased arterial pressure, abdominal adiposity and high systolic blood pressure. Furthermore, short maternal height was associated with a low birth weight offspring and stunting in children. Also in Brazil, Franco et al. [41] reported changes in the sympathoadrenal and renin-angiotensin systems in children born small for their gestational age. They investigated the plasma levels of angiotensin-converting enzyme (ACE), angiotensin, and catecholamine's in 8- to 13-year-old children to determine correlations between the plasma levels and both birth weight and blood pressure. Circulating noradrenaline levels were significantly elevated in small for gestational age girls compared to girls born with a weight appropriate for their gestational age. In addition, angiotensin II and ACE activity were higher in small for gestational age boys. There was a significant association between the circulating levels of both angiotensin II and ACE and SBP. Another study in Brazil [42] showed that ACE activity is increased, together with an increase in systolic and diastolic pressure, in children with stunting independent of birth weight. 

Although in Latin America the prevalence of type 2 diabetes mellitus in individuals that were undernourished in early life is not known, it is known that poor countries with an accelerated process of urbanization are particularly vulnerable and have been experiencing a considerable increase in diabetes prevalence [43]. Deleterious changes have been reported in glucose metabolism in Mexican children suffering from undernutrition in infancy. The above-mentioned study examined the effects of undernutrition in the first year of life on glucose tolerance and plasma insulin levels. These authors reported that early postnatal undernutrition was associated with an increased incidence of alterations in the adult life even after adjusting for differences in birth weight [43].

It is interesting to speculate that the increased rates of hypertension, metabolic syndrome, and type 2 diabetes mellitus, observed in low and middle income countries, could be the result of the discrepancy between the nutritional environmental during fetal and early life and the adult environment. This discrepancy causes a mismatch between the fetal programming of the subject and the adult circumstances created by the imposition of new life styles [44]. The conflict between the earlier programming and the later presence of abdominal obesity may have produced a higher sensitivity of this population to develop a state of low-degree inflammation, insulin resistance and, consequently, an epidemic of hypertension, metabolic syndrome, and diabetes. The relative roles played by genetic and environmental factors and the interaction between the two are still subjects of great debate and merit further research.

## 6. The Role of Angiotensin II and Adiponectin in Hypertension

The visceral adipocytes of people experiencing the rapid changes described above are overexpressing the gene that regulates the synthesis of angiotensin II (Ang II) [45]. Ang II is produced in adipocytes [45–47], and it has been demonstrated that plasma levels of angiotensinogen and Ang II are increased with an increase in BMI [47]. Ang II has three important effects in humans, which were crucial to survival when human beings were nomads, fruit collectors, hunters, and fishermen and endured long periods without food. (1) It blocks insulin intracellular signaling routes, as a mechanism to conserve blood glucose [48]; (2) it stimulates the production of aldosterone, maintaining plasma sodium and water [49]; (3) it stimulates the production of proinflammatory cytokines, such as TNF-alpha, to maintain an alert state to fight infections [50, 51].

Nowadays, however, the production of Ang II in visceral adipocytes appears to be harmful and the insulin resistance and the water retention produced by Ang II are associated with hypertension, especially in low and medium income countries where the excess of fast food and sedentary lifestyles are relatively recent [35]. Moreover, it appears that the adaptation to this situation in obese people of developed countries, which have had a longer period of adaptation to the Western lifestyle, is an overexpression of adiponectin which in contrast to Ang II improves the insulin sensitivity and has anti-inflammatory effects [52]. This may explain why there is a substantially higher proportion of obese people who are metabolically healthy in high income countries compared to low and middle income populations. We propose therefore that the increased production of Ang II and the decreased production of adiponectin in visceral fat were an appropriate human biological response to the conditions of limited access to food and water. However, nowadays the imposition of Western lifestyles, which the humans in underdeveloped countries are not particularly well adapted to, is the main cause of the alterations that are leading to the increased prevalence of hypertension [35].

Using segments of internal mammary arteries obtained from adults with severe coronary artery disease (CAD), we showed [53] that the presence of obesity was associated with a higher contractile response to Ang II, after matching for age, sex, glucose and insulin plasma levels, homeostatic model assessment (HOMA) index, lipid profile, tobacco and alcohol consumption, physical activity, and arterial blood pressure. Moreover, increased waist circumference was associated with progressively lower levels of adiponectin and higher levels of leptin in these patients. We observed significantly higher concentrations of CRP and IL-6 in dyslipidemic patients with a history of CAD compared to those without a history of CAD [54]. Elevated levels of these inflammatory markers were not associated with any further impairment of endothelial function, but they were associated with a higher carotid intima-media thickness (IMT) in those subjects with a previous history of CAD. These results suggest that in our population low grade inflammation is associated both with adiposity and with the progression of CAD. 

Ang II has been proposed as a trophic factor in white adipose tissue growth and development, since renin-angiotensin system components are influenced by nutritional state and adipose tissue mass [55–61]. Higher Ang II is also associated with lower birth weight [41]. Increased thermogenesis could also participate in the reduction of body weight, and Ang II seems to be also related to this effect [41]. The local renin-angiotensin system plays a role in adipocyte differentiation and in body-fat accumulation. In humans Ang II produced by mature adipocytes appears to inhibit the differentiation of adipocyte precursors, thus decreasing the percentage of small insulin-sensitive adipocytes and promoting the presence of large adipocytes [56–58], which decrease insulin sensitivity and produce ectopic deposition of lipids that promotes the development not only of hypertension, but also of insulin resistance and type 2 diabetes [62].

## 7. Conclusions

Hypertension, diabetes, and that cluster of metabolic alterations often referred to as the metabolic syndrome are highly prevalent in low and middle income countries which contribute to an increasing proportion of the worldwide burden of chronic disease. Ethnic differences in low grade inflammation are already evident in childhood and large epidemiological studies clearly show regional differences in the associations between AO, inflammation, and hypertension. While prevalence varies between countries within each region and within different areas in these countries [87], it appears that compared to developed countries populations within developing countries, have a greater susceptibility to hypertension and other cardiometabolic disease at a given level of adiposity. We argue that in low and middle income countries, elevated CRP (and other inflammatory markers) and angiotensin II, associated with the higher prevalence of maternal malnutrition and early growth restriction or childhood under nutrition, are important contributors to the higher susceptibility to hypertension and cardiovascular disease observed within these regions. In accordance, region-specific research is urgently needed to better understand interactions between genetic, epigenetic, and environmental factors operating in populations being rapidly exposed to Western lifestyles. Moreover, as recently we have showed, in our population nutritional intervention as a supplementation with aged garlic can increase the levels of adiponectin, probably contributing to improve the metabolic profile of Colombian subjects with abdominal obesity and hypertension [88].

## Figures and Tables

**Table 1:** Regional differences in the risk of stroke associated with hypertension (Adapted from: “O'Donnell, et al. [9]”).

Region	Risk of stroke associated with self-reported hypertension or blood pressure > 160/90 mm Hg (Odds-ratio)
Africa (*n* = 323)^*∧*^	4.96 (3.11–7.91)
Southeast Asia (*n* = 1146)^‡^	4.49 (3.54–5.70)
India (*n* = 958)	4.36 (3.34–5.69)
South America (*n* = 151)^†^	3.52 (1.63–7.60)
High income countries (*n* = 422)*	2.79 (1.83–4.25)

Data are odds ratio (99% CI). ^*∧*^Mozambique, Nigeria, South Africa, Sudan, and Uganda. ^‡^China, Malaysia, and Philippines. ^†^Argentina, Brazil, Chile, Colombia, Ecuador, and Peru. *Australia, Canada, Croatia, Denmark, Germany, Iran, and Poland.

**Table 2:** Ethnic differences in main pro-inflammatory cytokines.

Study (Author and Ref)	Subjects	Marker	Results	Comments
Albert et al. [63]	24,455 White, Hispanic, and Asian adult females. Health Study in the United States.	CRP	Median/interquartile range (IQR) Black: 2.96 mg/L (1.19, 5.86) White: 2.02 mg/L (0.81, 4.37) Hispanic: (2.06 mg/L (0.88, 4.88) Asian: (1.12 mg/L (0.48, 2.25)	Black women had significantly higher values of CRP than White, Hispanic, and Asian. *P* < 0.001

Chandalia et al. [64]	137 South Asian and White adult males	CRP	Mean: Asian Indians: 0.99 mg/dL White: 0.58 mg/dL	Asian Indians had significantly higher concentrations of hs-CRP than Caucasians. *P* = 0.036.

McDade et al. [65]	229 Black, White, and Hispanic adult.	CRP	Median/(IQR) White: Females: 1.05 mg/L (0.44, 1.88) Males: 0.59 (0.44, 1.50) Black: Females: 3.30 mg/L (1.39, 4.47) Males: 1.07 mg/L (0.37, 1.70) Hispanic: Females: 1.49 mg/L (0.78, 3.10) Males: 1.00 mg/L (0.55, 1.65)	Black had significantly higher CRP concentrations than the other groups. *P* = 0.007

Schutte et al. [66]	217 Black and White adult females. POWIRS study.	CRP	Mean ± SD Black: 4.59 mg/L (3.17; 6.01) White: 3.27 mg/L (2.56; 3.98)	Black women had significantly higher hs-CRP levels compared to white women. *P* < 0.05

Patel et al. [67]	1083 Black and White adults. Bogalusa Heart Study.	CRP	Mean ± SD White: Males: 1.8 ± 1.9 mg/L; Females: 2.5 ± 2.3 mg/L; Black: Males: 2.3 ± 2.3 mg/L Females: 2.7 ± 2.4 mg/L	Black had significantly higher CRP values than Whites. *P* < 0.01.

Khera et al. [68]	2,749 White and Black adults. Dallas Heart Study.	CRP	Median Black: 3.0 mg/L White: 2.3 mg/L;	Significantly higher CRP values in blacks. *P* < 0.001.

Ford et al. [69]	2205 Whites, Black, and Mexican American adult females. National Health and Nutrition Examination Survey 1999-2000.	CRP	Mean Whites: 2.3 mg/L Black: 3.1 mg/L Mexican American: 3.5 mg/L	Significantly higher CRP in Mexican-American women than White women. *P* < 0.001.

Ford et al. [70]	1940 White, Black, Mexican, and other American adults.	CRP	Median: White: 1.6 mg/L Black: 1.7 mg/L Mexican: 1.5 mg/L Other: 1.8 mg/L	No significant differences between ethnicities.

LaMonte et al. [71]	135 Black, Native and White adult females. American Cross-Cultural Activity Participation Study (CAPS).	CRP	Mean ± SD Native: 0.25 ± 0.03 mg/dL Whites: 0.23 ± 0.13 mg/dL Black: 0.43 ± 0.03 mg/dL	Significantly higher CRP concentrations among Black compared with Native and White. *P* = 0.002.

Elkind et al. [72]	279 Hispanic, Black, and White American adult. Northern Manhattan Stroke Study.	CRP	Mean ± SD White: 1.88 ± 2.75 mg/L Black: 2.64 ± 4.62 mg/L Hispanic: 2.11 ± 3.50 mg/L	There were some differences in levels of marker by ethnicity but none were statistically significant.
		TNF-*α*	White: 2.71 ± 4.25 pg/mL Black: 1.04 ± 1.63 pg/mL	
		IL-6	White: 1.15 ± 1.08 pg/mL Black: 1.36 ± 1.51 pg/mL	
		IL-1	White: 0.23 ± 0.43 pg/mL Black: 0.35 ± 0.59 pg/mL;	

Wener et al. [73]	22,000 multiethnic individuals age ≥ 4 yrs. Third National Health and Nutrition Evaluation Survey (NHANES III).	CRP	95th percentile value Males: 0.95 mg/dL Females: 1.39 mg/dL.	The values for Mexican-Americans and non-Hispanic whites were similar, compared with non-Hispanic black adults females, who had higher levels.

Chatha et al. [74]	191 White and Indo-Asian. British adults.	CRP	Mean ± SD Indo Asian: Female 2.29 (1.52) mg/L Male 1.77 (1.46) mg/ L Whites: Female 2.23 (1.54) mg/ L; Male 1.94 (1.45) mg/ L.	Serum CRP concentrations were similar in Indo-Asians and White.

Chambers et al. [75]	1532 Asians and White. British adults.	CRP	Mean ± SD: Whites: 1.47 ± 1.62 mg/L Asians: 1.71 ± 1.81 mg/L	Significantly higher CRP concentration in Asians compared with whites. *P* = 0.02.

Forouhi et al. [76]	113 adult South Asian and White British adults.	CRP	Mean White: Male: 0.92 (0.34–1.61) mg/L Female: 0.70 (0.41–1.70) mg/L South Asian: Male: 1.07 (0.76–1.50) mg/L Female: 1.35 (0.72–3.04) mg/L	Median CRP level in South Asian women was nearly double that in European women. (*P* = 0.05).
		CRP	Mean Black: 2.5 mg/L Whites: 2.1 mg/L	Afro-Caribbean had significantly higher TNF-*α* (*P* = 0.001), and IL-6 (*P* = 0.036) levels.

Kalra et al. [77]	160 Black and White. British adults.	IL-6	Whites: 1.5 pg/mL Black: 2.3 pg/mL	No significance in CRP levels despite elevated IL-6 and TNF-*α*.
		TNF-*α*	Whites: 4.3 ± 3.6 mg/m/L Black: 6.7 ± 6.1 pg/mL.	CRP was significantly lower in Black men and women than in other ethnic groups. *P* < 0.05.

Heald et al. [78]	440 White, Pakistani, and Black British adults. Population-based community survey.	CRP	Mean Black: Male: 1.0 mg/L Female: 1.3 mg/L White: Male: 2.2 mg/L Female: 2.1 mg/L Pakistani: Male: 1.7 mg/L Female: 2.8 mg/L 2.8(2.1–3.6) mg/L	

Mwantembe et al. [79]	72 Black and White adults. Study performed in South Africa.	IL-1	Mean ± SD Whites: 1.99 ± 1.88 pg/mL Blacks: 2.69 ± 2.58 pg/mL;	No significant differences

Petersen et al. [80]	482 South-Asians and White young adults.	IL-6 TNF- *α*	Mean: Whites: 0.78 pg/mL South-Asians: 1.60 pg/mL; Whites: 1.13 pg/mL South-Asians: 1.29 pg/mL	Significantly higher IL-6 concentrations in South-Asians compared with White men. *P* < 0.001.

Albandar et al. [81]	228 White, Hispanic, Black adults.	IL-1	Mean: White: 28.4 pg/mL Hispanic: 34.7 pg/mL Black: 21.7 pg/mL	Hispanics had higher IL-1beta concentrations than Blacks. *P* = 0.05.

Hong et al. [82]	70 White, Black American Adults.	IL-6	Mean: IL-6 1.36 (±0.80) pg/mL.	No significant differences between ethnicities.

**Table 3:** Pro-inflammatory cytokines in children and adolescents.

Study (Author and Ref)	Subjects	Marker	Results	Comments
López-Jaramillo, et al. [27]	325 schoolchildren (mean age, 10.0 years) from Colombia	CRP	Mean (mg/dL) ± SD Boys: 1.2 ± 2.6 Girls: 1.5 ± 2.0 BMI: 15 0.6 ± 0.9 BMI: 17 1.1 ± 2.2 BMI: 21 1.9 ± 3.7	CRP levels correlate significantly with BMI. (*P* < 0.01).

Gillum [83]	996 Mexican American children aged 6–11 years.	CRP	Detectable CRP was seen in 34.7% of overweight children but only 6.8% of other children (*P* = 0.0006, RR = 5.12, 95% CI: 3.32–7.90).	CRP levels correlate significantly with BMI. (*P* < 0.01).

Visser et al. [84]	3512 American children (8 to 16 years of age).	CRP	Percentile value CRP (mg/dL) CRP (4–11 years of age: >0.37 mg/dL for boys >0.68 mg/dL for girls Based on the BMI For overweight girls: 5.59 (95% CI: 2.20–14.22) For overweight boys: 6.12 (95% CI: 1.23–30.52)	CRP levels correlate significantly with BMI. (*P* < 0.01).

Cook et al. [28]	699 (10 to 11 years of age) multiethnic study in children.	CRP	Median mg/L All groups: 0.15 (IQ 0.06–0.47) South Asian: 2.40 (1.42, 4.04) Other: 0.82 (0.35, 1.87)	CRP was strongly related to adiposity (95% CI, 155–439%) and was higher in South Asian children.

Ford [29]	3348 White, Black and Mexican-American US children and young adults. National Health and Nutrition Examination Survey, 1999-2000,	CRP	Median White: 1.6 mg/L Black: 1.7 mg/L Mexican-American: 1.5 mg/L Other: 1.8 mg/L	No significant differences between ethnicities.

Aeberli et al. [30]	33 Swiss children (6 to 14 years of age). Normal-weight (*n* = 33), overweight (*n* = 19), and obese (*n* = 27)	CRP	CRP median (mg/dL) IL-6 (pg/mL) TNF-*α* (pg/mL) Overweight: 0.03 (0.01–0.42) Obese: 0.10 (0.03–0.23)	CRP, IL-6 increased significantly (*P* < 0.02) with increasing adiposity, independent of age.
		IL-6	Overweight: 0.34 (0.05–1.81) Obese: 0.41 (0.14–2.00)	
		TNF-*α*	Overweight: 6.3 (4.2–11.8) Obese: 7.2 (4.1–21.8)	

Weiss et al. [85]	439 White, Black and Hispanic, obese, overweight and nonobese American children and adolescents.	CRP	Mean CRP (mg/dL), IL-6 (pg/mL) Blacks moderately obese: 0.13 Severely obese: 0.32 Whites moderately obese: 0.12 Severely obese: 0.31 Hispanics moderately obese: 0.13 Severely obese: 0.35	Interleukin-6 and CRP were significantly related to the degree of obesity (*P* < 0.001)
		IL-6	Blacks moderately obese: 1.89 Severely obese: 2.36 Whites moderately obese: 1.59 Severely obese: 1.80 Hispanics moderately obese: 2.07 Severely obese: 3.09	

Vikram et al. [86]	62 Indian adolescents	CRP	Mean (mg/dL) ± SD Normal weight: 2.5 ± 2.7 Overweight: 4.1 ± 2.4	CRP levels correlate significantly with BMI (*P* < 0.05).
